# Dapagliflozin mitigates oxidative stress, inflammatory, and histopathological markers of aging in mice

**DOI:** 10.25122/jml-2023-0343

**Published:** 2024-02

**Authors:** Elaf Mahmood Shihab, Haitham Mahmood Kadhim, Samer Salim Shahooth

**Affiliations:** 1 Department of Pharmacology, College of Pharmacy, Al-Esraa University, Baghdad, Iraq; 2 Department of Clinical Pharmacy, College of Pharmacy, Al-Nahrain University, Baghdad, Iraq; 3 Department of Pharmacology, College of Health and Medical Technology, Uruk University, Baghdad, Iraq

**Keywords:** aging, skin, heart, inflammation, oxidative stress, Dapagliflozin, AGEs, Advanced Glycation End Products, Ca2+, Calcium, CVD, Cardiovascular Disease, Col-I, Collagen I, Col-III, Collagen III, DAPA, Dapagliflozin, GSH-Px, Glutathione Peroxidase, H&E, Hematoxylin and Eosin Stain, HPF, High Power Fields, IL-1β, Interleukin-1 Beta, IP, Intraperitoneally, MDA, Malondialdehyde, ROS, Reactive Oxygen Species, SGLT2i, Sodium-Glucose Cotransporter 2 Inhibitors, SGLT2, Sodium-Glucose Cotransporter-2, SD, Standard Deviation, TNF-α, Tumor Necrosis Factor-Alpha

## Abstract

Aging, a complex physiological process affecting all living things, is a major area of research, particularly focused on interventions to slow its progression. This study assessed the antiaging efficacy of dapagliflozin (DAPA) on various aging-related parameters in a mouse model artificially induced to age. Forty male Swiss albino mice were randomly divided into four groups of ten animals each. The control group (Group I) received normal saline. The aging model group (Group II) was administered D-galactose orally at 500mg/kg to induce aging. Following the aging induction, the positive control group received Vitamin C supplementation (Group III), while the DAPA group (Group IV) was treated with dapagliflozin. The inflammatory mediators (TNF-α and IL-1β) showed similar patterns of change. No statistically significant difference was observed between groups III and IV. Both groups had significantly lower values compared to GII, while it was significantly higher compared to GI. Glutathione peroxidase (GSH-Px) showed no statistically significant difference between groups GIII and GIV, but it was higher in GIII compared to GII and significantly lower in GIII compared to GI. The study demonstrated that dapagliflozin exerts a beneficial impact on many indicators of aging in mice. The intervention resulted in a reduction in hypertrophy in cardiomyocytes, an enhancement in skin vitality, a decrease in the presence of inflammatory mediators, and an improvement in the efficacy of antioxidants.

## INTRODUCTION

Aging is a natural physiological process characterized by progressive changes at the cellular level. These changes impact the structure and function of cells and their surrounding environment. Injuries and accumulated damage throughout life contribute to this decline, gradually disrupting the body's regulatory systems [1]. There are several prevalent characteristics of aging in mammals. Firstly, there is a noticeable rise in mortality rates after reaching maturity [2]. Additionally, there are notable alterations in the biochemical makeup of tissues as mammals age. These changes often manifest as decreased muscle mass and total density of bones in humans and the accumulation of lipofuscin, a pigment associated with aging [3]. Various factors contribute to aging, but the primary factor is the progressive buildup of molecular damage that occurs randomly and remains unrepaired. This damage disrupts cellular functions and can lead to abnormalities, ultimately impacting tissue health and promoting aging [4]. Some mechanisms involved include genomic instability [5], telomere attrition [6], epigenetic alterations [7], and loss of proteostasis [8]. These processes operate within a multi-layered model, working in conjunction to ultimately contribute to the progression of the aging process [9]. Physiological aging significantly influences most biological systems in the human body [10]. The impact of aging on the skin has been a significant topic of discourse across various disciplines for an extended period [11]. The process of skin aging can be classified into two categories: intrinsic aging, which occurs chronologically from within the body, and extrinsic aging, which is attributed to external influences [12].

Dapagliflozin (DAPA) is a novel antihyperglycemic drug that selectively blocks the sodium-glucose cotransporter-2 (SGLT2) in a competitive and reversible manner. It belongs to a recent class of medications used for managing type 2 diabetes. The inhibition of SGLT2 by DAPA results in a reduction of glucose reabsorption into the systemic circulation. This leads to increased glucose filtration via the renal system and subsequent excretion in the urine, ultimately decreasing glucose levels. Notably, this effect is independent of the action of insulin [13-15]. DAPA has also been recognized for its antioxidant properties, offering cellular protection by scavenging oxidative free radicals. This reduces oxidative damage by modulating the function or production of pro-oxidant enzymes, such as Nox4, endothelial nitric oxide synthase, and xanthine oxidase [16]. DAPA has anti-inflammatory properties by reducing the amount of inflammatory cytokines mRNA, such as interleukin-1 beta (IL-1β) and IL-6. Additionally, it enhances the expression of anti-inflammatory cytokines mRNA, such as IL-10, and promotes a boost in the ratio of M2/M1 phenotypic macrophages. The M1 phenotype of macrophages is characterized by its pro-inflammatory properties, while the M2 phenotype exhibits anti-inflammatory characteristics [17]. This study aimed to evaluate the effectiveness of DAPA in mitigating age-related changes in mice with induced aging by analyzing various physiological parameters.

## MATERIAL AND METHODS

### Study design

**Figure 1:**
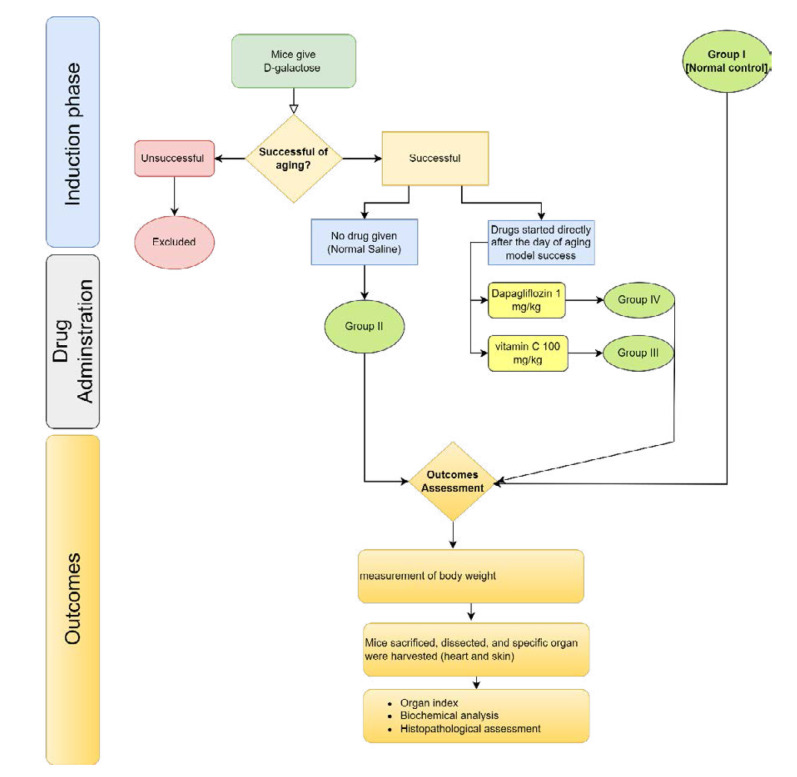
Study flowchart

Forty male Swiss albino mice, aged 3-6 months and weighing 20-30 grams, were obtained from the National Center for Drug Control and Research and housed in polypropylene cages. These mice were randomly divided into four groups, each comprising 10 mice. Individual mice were identified by labeling different body parts. The housing conditions were maintained at 22 ± 2 °C, with an inverted 12-hour light-dark cycle. Before the experiment began, mice underwent a two-week acclimation period at the Animal Facility of Al-Nahrain University - Biotechnology Research Center, Baghdad, Iraq. During this time, mice were provided with a standard pellet diet and unlimited access to water. Details of the animal allocation are provided in Table 1 and illustrated in Figure 1 [18-22].

**Table 1:** Animal treatment groups

	D-galactose^a^	Drug received	Duration
Group I [18]	No	Normal saline by gastric gavage	6 weeks
Group II [19]	Yes	-	6 weeks
Group III [18,20]	Yes	Vitamin C (100 mg/kg)	6 weeks
Group IV [21,22]	Yes	DAPA (1 mg/kg)	6 weeks

a dosage of 500mg/kg of D-galactose orally by gastric gavage for 6 weeks

### Preparation of compounds

D-Galactose (Sigma Aldrich, CAS No. 59-23-4), DAPA (Hangzhou Hyper Chemicals Limited, CAS No. 461432-26-8), and Vitamin C (Hangzhou Hyper Chemicals Limited, CAS No. 86404-04-8) were used.

### Induction and assessment of aging in mice

**Figure 2:**
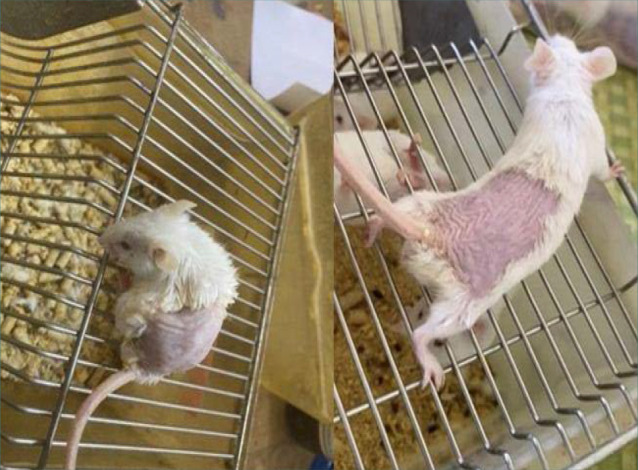
Induction of the aging process in mice

To induce an aging phenotype, 30 male Swiss albino mice were administered D-galactose (500 mg/kg body weight) orally via gastric gavage for six weeks. Mice were monitored for phenotypic changes associated with aging, such as ruffled fur, rounded body shape, decreased alertness and activity, and skin wrinkling. Additionally, their movements were observed for reduced responsiveness or increased caution compared to younger mice (Figure 2) [23]. Mice not exhibiting these aging characteristics were excluded to ensure a consistent aging model across the study.

### Sample size and randomization

The sample size for this study was calculated using the G*Power program [24] based on Cohen's principles [25]. The randomization into different experimental groups was achieved using a table of random integers to prevent selection bias. Each mouse was systematically placed in a specifically labeled container to facilitate clear identification and avoid confusion, and a unique tail tag was given [26].

### Tissue collection and analysis

The weight of all mice was recorded at the start and end of the study. After a 12-week treatment period and following a 12-hour fasting period, mice were anesthetized with a combination of 80 mg/kg of ketamine and 10 mg/kg xylazine administered intraperitoneally (IP). Complete anesthesia was confirmed before euthanasia by exsanguination via heart puncture, a method suitable for subsequent tissue collection and preservation [27,28]. Post-euthanasia, the mice were dissected, and the hearts were excised and weighed to calculate the organ index, utilizing the equation as described by Chen *et al*. [29].


$$Organ\;index(\%)=\frac{organ\;weight(g)}{body\;weight(g)}x100\%$$

A 1 mm-thick skin segment was also collected from the dorsal region. The heart tissue was divided into two sections. A single specimen was employed for histological examination. This specimen was initially rinsed with phosphate-buffered saline (PBS) at a pH of 7.4 and prepared using the paraffin-embedding technique [30]. The additional heart and skin tissue was isolated and subjected to a cold phosphate buffer saline (PBS, pH 7.4) rinse. Subsequently, the tissue was dried using filter paper and utilized for ELISA analysis using an ELISA reader from Diagnostic Automation/Cortez Diagnostics. Additionally, the tissue was weighed using a sensitive balance. For the ELISA, 50 mg of each tissue was placed in an Eppendorf tube with 0.45 ml of cold PBS. The tissues were then mechanically homogenized using an electric tissue homogenizer (Staruar) while maintaining the sample on ice. Subsequently, the tissue was finely chopped into small fragments and placed in a beaker containing ice to maintain a low temperature. The sample was homogenized using an electrical tissue homogenizer (Staruar). The resulting homogenate was centrifuged at 4°C and 2000 rpm for 20 minutes using a cold centrifuge (Thermos Scientific). The supernatant was extracted utilizing a micropipette manufactured by Bioevopeak and preserved at a temperature of -20°C for future analysis [31].

### Assessment of hypertrophic cells in heart tissue

Five cross-sectional heart tissue images were captured for each mouse using a digital camera attached to a light microscope (BX-FLA; Olympus). A histopathologist assessed the presence of hypertrophic cells, counting their number across five high power fields (HPF) at 400x magnification, corresponding to an approximate area of 1 mm^2^. This method, adapted from Cree *et al*. and based on the World Health Organization (WHO) classification of tumors, involved enumerating hypertrophic cells characterized by increased size, bizarre shapes, irregular and hyperchromatic nuclei, as seen in H&E-stained sections. The total cell count across these fields was considered the hypertrophic cell count for each specimen.

### Biochemical analysis

The supernatants previously stored from homogenized heart and skin tissues were thawed for biochemical assays. The concentrations of tumor necrosis factor-alpha (TNF-α), interleukin-1 beta (IL-1β), glutathione peroxidase (GSH-Px), malondialdehyde (MDA), collagen I (Col-I), and collagen III (Col-III) were measured using the double-sandwich ELISA technique. Specific ELISA kits from Sunlong Biotech were employed for each biomarker (Product IDs: TNF-α SL0547Mo, IL-1β SL0316Mo, GSH-Px SL0241Mo, MDA SL0370Mo, Col-I SL0141Mo, Col-III SL0942Mo).

### Light microscopy

Cardiac myocyte morphology was examined using an Olympus BX51 Microscope (Olympus Corporation). Observations were made randomly across five different zones per slide, including the corners and center, at a 40x magnification level.

### Statistical analysis

Data analysis was performed using GraphPad Prism version 10.0.1, presenting descriptive statistics as mean ± standard deviation (SD). The Shapiro-Wilk test confirmed that all variables were normally distributed. Differences among groups were evaluated using one-way ANOVA, with post hoc comparisons made via Tukey's test. A *P* value of ≤0.05 was considered statistically significant.

## RESULTS

### Inflammatory mediators and oxidative stress markers

Inflammatory mediators, including TNF-α, IL-1β, and MDA, were significantly elevated in the D-galactose-induced aging group (GII) compared to the control group (GI) (*P* <0.0001). No statistically significant difference was observed between groups GIII and GIV. However, GIII and GIV had significantly lower values than GII and significantly higher values than GI. The activity of glutathione peroxidase (GSH-Px) was significantly lower in the induction group (GII) compared to the control group (GI) (*P* <0.0001). There was no statistically significant difference between GIII and GIV. However, the mean value of GIII was significantly greater than that of GII but significantly lower than that of GI (Table 2).

**Table 2:** Effect of DAPA and Vitamin C on inflammatory mediators and oxidative stress markers

Groups	TNF-α Mean ± SD	IL-1β Mean ± SD	GSH-Px Mean ± SD	MDA Mean ± SD
GI: Normal control	27.70 ± 1.57^a^	16.08 ± 3.17^a^	5.10 ± 0.53^a^	22.92 ± 7.72^a^
GII: Induction	299.60 ± 92.96^b^	68.40 ± 6.20^b^	0.29 ± 0.15^b^	217.15 ± 55.25^b^
GIII: Vitamin C 100 mg/kg after end of induction	87.66 ± 8.64^c^	29.42 ± 4.42^c^	3.86 ± 0.54^c^	56.60 ± 7.94^c^
GIV: DAPA 1 mg/kg after end of induction	91.52 ± 8.87^c^	30.69 ± 4.52^c^	3.59 ± 0.75^c^	62.30 ± 9.92^c^
*P* value	<0.0001^***#^	<0.0001^***#^	<0.0001^***#^	<0.0001^***#^

Columns with similar letters indicate no significant difference (P value ≥0.05), while different litters indicate a significant difference (*P* value <0.05)

### Cardiac and skin biomarkers

The mean organ index of the heart and the count of hypertrophy cells in the heart were significantly higher in the induction group (GII) compared to the control group (GI) (*P* <0.001). There was no statistically significant difference between GIII and GIV, although both showed lower values than GII and higher than GI (Table 2). Skin collagen markers COL-1 and COL-III were significantly decreased in GII compared to GI (*P* <0.0001), with GIII showing higher levels than GIV (Table 3).

**Table 3:** Evaluation of heart organ index and skin collagen

Groups	COL-1 Mean ± SD	COL-III Mean ± SD	Heart hypertrophic cell count Mean ± SD	Heart index Mean ± SD
GI: Normal control	3,095.34 ± 295.70^a^	2,895.34 ± 295.70^a^	21.70 ± 2.54^a^	0.35 ± 0.11^a^
GII: Induction	837.47 ± 244.55^b^	637.47 ± 244.55^b^	90.30 6.73^b^	0.83 ± 0.05^b^
GIII: Vitamin C 100mg/kg	2,527.16 ± 323.56^c^	2,127.16 ± 323.56^c^	56.10 ± 5.67^c^	0.55 ± 0.07^c^
GIV: DAPA 1 mg/kg	1,756.86 ± 171.06^d^	1,556.86 ± 171.06^d^	59.20 ± 6.55^c^	0.57 ± 0.07^C^
*P* value	<0.0001^***#^	<0.0001^***#^	<0.0001^***#^	<0.0001^***#^

Columns with similar letters indicate no significant difference (P value ≥0.05), while different letters indicate a significant difference (*P* value <0.05)

### Histological observations

**Figure 3:**
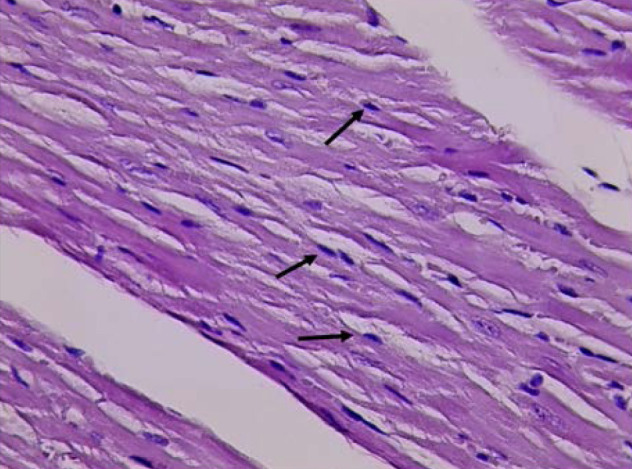
Histological evaluation of cardiac tissue from Group I (H&E stain). Cardiac tissue section from Group I (treated with normal saline only) observed under light microscopy (Olympus BX51 microscope with DP controller software at 40x magnification). The image shows typical cardiac cell morphology with normal nuclei (indicated by black arrows).

**Figure 4:**
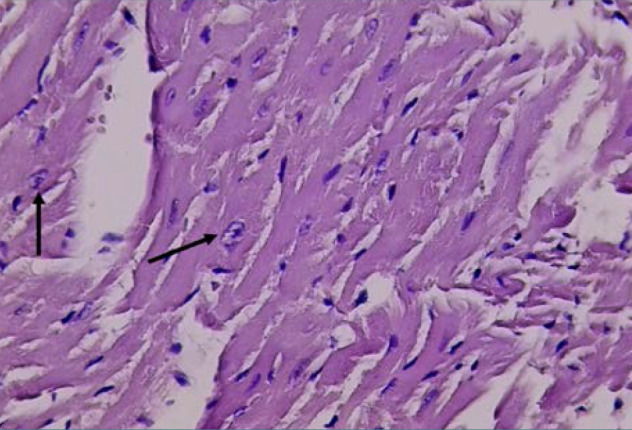
Histological evaluation of cardiac tissue from Group II (H&E stain). Cardiac tissue section from Group II (treated with D-galactose only) observed under light microscopy (Olympus BX51 microscope with DP controller software at 40x magnification). The image highlights abnormal cardiac cells with irregular and hyperchromatic nuclei (indicated by black arrows).

**Figure 5:**
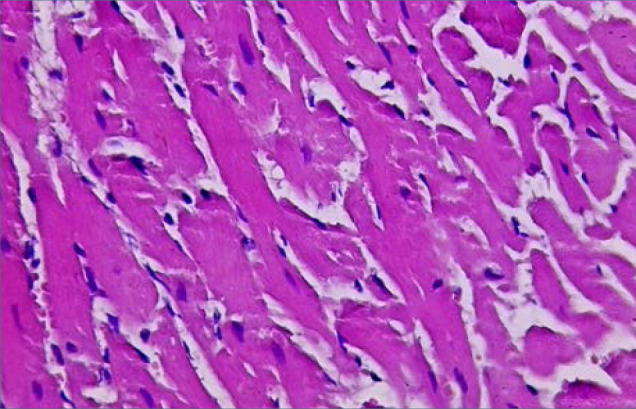
Histological evaluation of cardiac tissue section from Group IV (H&E stain). Cardiac tissue section from Group IV (treated with 1 mg/kg DAPA) observed under light microscopy (Olympus BX51 microscope with DP controller software at 40x magnification). The section displays normal cardiac cells and nuclei, comparable to the control group.

Histological analysis confirmed normal cardiac cell morphology in the control group (GI) with normal nuclei (Figure 3). In contrast, the D-galactose group (GII) displayed irregular and hyperchromatic nuclei (Figure 4). The DAPA group (GIV) returned to normal cell morphology and nuclei (Figure 5).

## DISCUSSION

In the current investigation, D-galactose administration induced cardiac hypertrophy, an effect mitigated by treatment with DAPA, as evidenced by a reduced heart index compared to the D-galactose-only group. This is in line with findings from Refaie *et al*. [32], where DAPA was shown to counteract the increase in heart weight caused by Cadmium toxicity and further supported by research indicating the efficacy of DAPA in reducing myocardial hypertrophy [33].

Multiple studies have demonstrated that this drug category can potentially improve cardiac morphological alterations, such as heart hypertrophy and fibrosis. Sodium-glucose cotransporter 2 inhibitors (SGLT2i) have been observed to reduce both cardiac preload and afterload by reducing intracellular sodium (Na+) and calcium (Ca2+) loading. This suggests that DAPA, a specific SGLT2i, may have a preventive effect on cardiac hypertrophy [34]. One probable mechanism is that DAPA plays a positive role in cardiomyocytes by upregulating the expression of Sirtuin 1 (SIRT1) [35]. In cardiomyocytes, nuclear SIRT1 blocks myocyte injury from oxidative stress by enhancing the expression of manganese superoxide dismutase (MnSOD) and increasing the expression of antioxidants, such as catalase. Studies have shown that overexpression of SIRT1 in the heart can attenuate age-dependent increases in cardiac hypertrophy [36,37].

One of the most significant alterations with age is a deregulation of the immune response, resulting in a chronic systemic inflammatory state [38]. This study evaluated various inflammatory markers in heart tissue homogenates from different treatment groups. DAPA treatment, both during and after the induction phase of aging, significantly reduced the levels of TNF-α and IL-1β compared to the control group. These findings align with previous research by ElMahdy *et al*. [39], who demonstrated that DAPA treatment in rats fed a high-carbohydrate, high-fat diet (HFHC) significantly reduced TNF-α and IL-1β levels compared to the HFHC group alone. Similarly, Chen *et al*. [40] revealed that mice treated with DAPA showed dramatic reduction in blood and cardiac levels of IL-1β, TNF-α, and IL-6 levels compared to untreated mice with diabetes.

DAPA administration has been shown to reduce excess calcium, thereby mitigating inflammation and decreasing the levels of various pro-inflammatory cytokines, including those involved in the IL-1β pathway [41]. It also shifts M1 macrophages, typically associated with promoting inflammation, towards M2 macrophages, which are involved in healing and regeneration. These findings indicate that DAPA, independent of glucose concentrations, exerts direct anti-inflammatory effects by suppressing toll-like receptor 4 (TLR-4) and nuclear factor kappa-light-chain-enhancer of activated B cells (NF-kB) activation and the secretion of pro-inflammatory mediators [42]. In addition, DAPA therapy increased IL-10 levels, which controls both acute and chronic inflammation by inhibiting the production of proinflammatory cytokines from immune cells such as TNF-α [43].

In this study, we assessed oxidative stress markers in heart tissue homogenates across various treatment groups. The administration of DAPA was associated with increased levels of GSH-Px and decreased levels of MDA, indicative of reduced oxidative stress compared to the D-galactose-induced aging group. Supporting our findings, other research has demonstrated that DAPA significantly lowered MDA levels in cardiac tissue homogenates from diabetic animals compared to untreated diabetic animals. Moreover, unlike the untreated diabetes mellitus (DM) group, the groups treated with DAPA had a significant increase in the antioxidant glutathione peroxidase (GPx) [44]. A study by Kıngır *et al*. consistently reported that DAPA administration decreased MDA levels and increased GSH levels, aligning with our results [45].

SGLT2 inhibitors are crucial in maintaining a healthy redox balance, protecting mitochondria from dysfunction. They also contribute to normoglycemia, which lowers advanced glycation end-products (AGE) levels. AGEs are a significant source of free radical production, largely because hyperglycemia strongly triggers their formation and amplifies the interaction between AGEs and their receptor (RAGEs), enhancing oxidative stress [46]. In addition, SGLT2 inhibitors have been shown to reduce insulin resistance, which is closely associated with oxidative stress in diabetes, which suggests that they may indirectly reduce oxidative stress [47,48].

In the present study, DAPA administration, concurrent with or after D-galactose-induced aging, significantly elevated the skin collagen types COL-I and COL-III. These findings are supported by Horikawa *et al*. [49], who found that diabetic mice treated with DAPA exhibited higher levels of total skin collagen than those untreated, suggesting a protective role of DAPA against collagen degradation. The underlying mechanism may involve the inhibition of mast cell activation and subsequent matrix metalloproteinase-1 (MMP-1) secretion, both involved in collagen breakdown [49].

Accumulating reactive oxygen species (ROS) from free radicals is widely believed to significantly cause skin aging [50]. Mitogen-activated protein kinase (MAPK) activation is a common consequence of elevated ROS generation. Collagen production declines with aging because MAPK activation activates activated protein-1 (AP-1), which then increases the expression of matrix metalloproteinases (MMPs) [51]. Long-term oxidative damage to cells and tissues is a key factor in the aging process, highlighting the potential for intervention strategies aimed at mitigating the detrimental effects of aging [52]. We observed a significant reduction in the levels of GSH-Px in mice treated with DAPA, while MDA was significantly increased in mice treated with DAPA, which partly explains the antiaging effect of DAPA.

The current study showed that the heart hypertrophic cell count was significantly increased in the induction group compared to the control group and other treated groups. Additionally, all these groups were significantly different compared to induction and normal groups. Research conducted by Yango *et al*. [35] showed that DAPA treatment significantly reduced cardiac hypertrophy in mice induced with Angiotensin II. Similarly, another study highlighted that pretreatment with the SGLT2 inhibitor DAPA decreased myocardial hypertrophy in rats infused with Angiotensin II [53].

Pro-inflammatory cytokines, such as TNF-α and IL-1β, cause cardiac myocyte hypertrophy, contractile dysfunction, and left ventricular dilatation and modulate the interstitial matrix of the heart. Our findings support the anti-inflammatory role of SGLT2i as a possible mechanism of action in the prevention of cardiovascular disease (CVD) [54]. The anti-hypertrophic effect of DAPA and carvedilol may be attributed to their anti-inflammatory properties. This is in line with several studies that reported elevated levels of circulating inflammatory markers, including tumor necrosis factor and IL-6, in hypertrophic cardiomyopathy (HCM), supporting the anti-inflammatory role of these treatments in cardiac health [55,56].

### Limitations

While animal models provide valuable insights into human diseases, they may not entirely replicate human pathologies. The study did not include clinical data from human subjects. Although the results in the mouse model are promising, further research is needed to determine the safety and efficacy of investigated drugs in humans.

## CONCLUSION

DAPA had a positive effect on several aging parameters in mice, as shown by this study. It decreased the hypertrophy in cardiomyocytes, improved skin vitality, decreased the burden of inflammatory mediators, and improved the impact of antioxidants.

## Data Availability

Zenodo: “Antiaging activity of dapagliflozin” [57]. https://doi.org/10.5281/zenodo.8267157
